# Sodium-Glucose Co-Transporter 2 Inhibitor Use and Risk of Major Adverse Cardiovascular Events in Patients With Rheumatoid Arthritis and Type 2 Diabetes: A Retrospective Observational Cohort Study Using Real-World Data

**DOI:** 10.14740/jocmr6577

**Published:** 2026-05-31

**Authors:** Meet Popatbhai Kachhadia, Piyush Puri, Krina Patel, Usmaan Topiwala, Juber D. Shaikh, Nirali Borad, Gurnoor Gill, Samarth Shah, Jay Patel, Harshal A. Sanghvi

**Affiliations:** a Department of Neurology, Charles E. Schmidt College of Medicine, Florida Atlantic University, Boca Raton, FL, USA; b Department of Internal Medicine, Icahn School of Medicine at Mount Sinai, Queens, New York, NY, USA; c Department of Internal Medicine, Charles E. Schmidt College of Medicine, Florida Atlantic University, Boca Raton, FL, USA; d SMT NHL Municipal Medical College, Ahmedabad, Gujarat, India; e Department of Neurology, Henry Ford Hospital, Detroit, MI, USA; f WVU Thomas Memorial Hospital, Charleston, WV, USA; g Department of Technology and Clinical Trials, Advanced Research LLC, Pompano Beach, FL, USA; h Department of Biomedical Engineering, Florida Atlantic University, Boca Raton, FL, USA; i Department of Information Technology and Operations Management (ITOM), College of Business, Florida Atlantic University, Boca Raton, FL, USA

**Keywords:** Major adverse cardiovascular events, Propensity score matching, Real-world evidence, Rheumatoid arthritis, SGLT2 inhibitors, Type 2 diabetes mellitus

## Abstract

**Background:**

Patients with rheumatoid arthritis (RA) and type 2 diabetes mellitus (T2DM) face elevated cardiovascular risk. Sodium-glucose co-transporter 2 (SGLT2) inhibitors have demonstrated cardiovascular benefits in diabetic populations, but their effectiveness in RA patients with T2DM remains inadequately explored. The study aimed to compare the risk of major adverse cardiovascular events (MACEs) between SGLT2 inhibitor users and dipeptidyl peptidase-4 (DPP-4) inhibitor users among patients with concurrent RA and T2DM.

**Methods:**

This retrospective cohort study utilized the TriNetX US Collaborative Network database, encompassing 67 healthcare organizations. We identified adult patients with documented RA and T2DM prescribed either SGLT2 inhibitors (n = 823) or DPP-4 inhibitors without SGLT2 inhibitor exposure (n = 284) between 2006 and 2026. Propensity score matching (1:1) balanced baseline demographics and comorbidities, yielding 277 matched pairs. The primary outcome was a composite MACE endpoint comprising myocardial infarction, cerebral infarction, heart failure, cardiovascular death, and cardiac arrest. Kaplan-Meier survival analysis and Cox proportional hazards regression were employed to assess time-to-event outcomes.

**Results:**

After propensity score matching, baseline characteristics were well-balanced between cohorts (standardized differences < 0.2). The SGLT2 inhibitor cohort showed a numerically lower, but statistically non-significant, MACE risk (43.0% vs. 48.7%; risk difference −5.8%, 95% confidence interval (CI): −14.1% to 2.5%; P = 0.172; odds ratio 0.792, 95% CI: 0.567– 1.107). Median survival time was 2,227 days in the SGLT2 inhibitor group versus 2,750 days in the DPP-4 inhibitor group. Kaplan-Meier analysis showed a borderline difference in event-free survival (log-rank P = 0.051), though interpretation is limited by substantially different follow-up durations between groups (median 3.1 vs. 6.4 years). However, the Schoenfeld residual test indicated significant violation of the proportional hazards assumption (P = 0.026), rendering the Cox hazard ratio of 1.284 (95% CI: 0.998–1.652) non-interpretable as a summary measure of treatment effect.

**Conclusions:**

Among patients with RA and T2DM, SGLT2 inhibitor use was associated with a numerically lower but statistically non-significant incidence of MACE compared to DPP-4 inhibitors. These findings are hypothesis-generating and do not provide statistically significant evidence of cardiovascular benefit. The lack of significance may reflect limited statistical power (277 matched pairs) and substantial differential follow-up duration between groups. Adequately powered prospective studies with standardized follow-up periods and comprehensive adjustment for RA-specific confounders are needed to determine whether SGLT2 inhibitors confer cardiovascular benefit in this high-risk population.

## Introduction

### Cardiovascular risk in rheumatoid arthritis (RA)

RA is a chronic systemic inflammatory disease affecting approximately 1% of the global population, characterized by symmetric polyarthritis, systemic inflammation, and substantially increased cardiovascular morbidity and mortality [1]. The inflammatory milieu in RA, driven by pro-inflammatory cytokines such as tumor necrosis factor-alpha (TNF-α), interleukin-6 (IL-6), and interleukin-1 beta (IL-1β), accelerates atherosclerosis through multiple mechanisms including endothelial dysfunction, oxidative stress, and dyslipidemia [2, 3]. This chronic systemic inflammation elevates cardiovascular disease (CVD) risk independent of traditional risk factors, with RA patients experiencing a 1.5- to 2-fold increased risk of myocardial infarction and heart failure compared to the general population [4].

Multiple longitudinal studies have demonstrated that the magnitude of cardiovascular risk in RA is comparable to that observed in type 2 diabetes mellitus (T2DM), establishing RA as an independent CVD risk equivalent [5, 6]. This elevated risk persists even after adjustment for traditional cardiovascular risk factors, suggesting that systemic inflammation represents an independent contributor to cardiovascular pathology [5]. Furthermore, patients with RA exhibit accelerated subclinical atherosclerosis, demonstrated by increased carotid intima-media thickness and coronary artery calcification, often preceding clinical cardiovascular events [7].

### The convergence of RA and T2DM

T2DM frequently coexists with RA, with prevalence rates ranging from 15% to 30% in RA populations significantly higher than age-matched controls [8, 9]. Multiple pathogenic mechanisms link these conditions, including shared inflammatory pathways involving the JAK/STAT signaling cascade, NLRP3 inflammasome activation, and cytokine-mediated insulin resistance [10]. Chronic elevation of pro-inflammatory cytokines, particularly IL-6 and TNF-α, impairs insulin signaling, promotes β-cell dysfunction, and contributes to progressive glucose dysregulation [10].

This dual diagnosis creates a synergistic cardiovascular risk profile, as both conditions independently contribute to endothelial dysfunction, oxidative stress, and accelerated atherosclerosis [3, 10]. Patients with concurrent RA and diabetes or insulin resistance demonstrate the highest hazard ratios (HRs) for developing CVD, with some studies reporting HRs exceeding 2.5 compared to the general population [6]. The intersection of chronic inflammation from RA and metabolic dysregulation from T2DM necessitates careful selection of glucose-lowering agents that not only achieve glycemic control but also mitigate cardiovascular risk.

### Sodium-glucose co-transporter 2 (SGLT2) inhibitors and cardiovascular protection

SGLT2 inhibitors represent a novel class of anti-hyperglycemic agents that reduce plasma glucose through increased urinary glucose excretion by inhibiting glucose reabsorption in the proximal renal tubule. Beyond glycemic benefits, landmark cardiovascular outcome trials have demonstrated robust cardiovascular and renal protective effects in T2DM populations with established CVD or multiple risk factors [11–13].

The EMPA-REG OUTCOME trial demonstrated a 14% reduction in the primary composite major adverse cardiovascular event (MACE) endpoint with empagliflozin (HR 0.86, 95% confidence interval (CI) 0.74–0.99) and notably a 38% reduction in cardiovascular death [11, 14].

Similarly, the CANVAS Program showed a 14% MACE reduction with canagliflozin (HR 0.86, 95% CI 0.75–0.97) [12, 15].

The DECLARE-TIMI 58 trial demonstrated a 17% reduction in the composite of cardiovascular death or heart failure hospitalization with dapagliflozin (HR 0.83, 95% CI 0.73–0.95) [13, 16].

The DAPA-HF trial extended these benefits to heart failure patients regardless of diabetes status [17], and recent meta-analyses confirm significant reductions in all-cause mortality and cardiovascular events across diverse populations [18, 19].

These cardiovascular benefits are mediated through pleiotropic mechanisms including hemodynamic, metabolic, anti-inflammatory, and renal protective effects, which may be particularly relevant in RA populations with chronic inflammation [18, 20].

### Dipeptidyl peptidase-4 (DPP-4) inhibitors as active comparator

DPP-4 inhibitors represent another established glucose-lowering class that enhances incretin hormone activity by inhibiting DPP-4-mediated degradation of glucagon-like peptide-1 (GLP-1) and glucose-dependent insulinotropic polypeptide (GIP). Large cardiovascular outcome trials including SAVOR-TIMI 53 (saxagliptin), EXAMINE (alogliptin), and TECOS (sitagliptin) have demonstrated cardiovascular safety, establishing non-inferiority to placebo for MACEs [21]. However, unlike SGLT2 inhibitors, DPP-4 inhibitors have not demonstrated consistent cardiovascular benefit signals in most trials.

DPP-4 inhibitors serve as an appropriate active comparator in this study, representing another second-line glucose-lowering option with established cardiovascular safety but without the demonstrable cardiovascular benefits of SGLT2 inhibitors [22, 23].

### Evidence gap in RA populations

Despite compelling evidence for SGLT2 inhibitor cardiovascular benefits in general T2DM populations, data regarding their efficacy and safety specifically in patients with concurrent RA and T2DM remain scarce. The unique inflammatory profile of RA, characterized by persistent elevation of pro-inflammatory cytokines, along with frequent corticosteroid use and disease-modifying antirheumatic drug (DMARD) therapies, may modulate SGLT2 inhibitor effects through several mechanisms [2, 3].

Chronic systemic inflammation, immunosuppressive therapies (corticosteroids, DMARDs, biologics), and RA-associated metabolic alterations may modulate SGLT2 inhibitor effects in ways not captured by existing trial data.

Importantly, RA populations have been systematically excluded or underrepresented in landmark cardiovascular outcome trials of glucose-lowering agents, creating a critical evidence gap for clinical decision-making in this high-risk population [11–13]. The intersection of chronic inflammatory disease and metabolic dysregulation represents a burgeoning area requiring dedicated investigation to optimize therapeutic strategies.

### Study objectives

The primary objective of this study was to compare the incidence of MACEs between SGLT2 inhibitor users and DPP-4 inhibitor users among patients with concurrent RA and T2DM, utilizing real-world evidence from a large federated health research network. Secondary objectives included assessment of individual MACE components and evaluation of time-to-event outcomes through survival analysis. We hypothesized that SGLT2 inhibitor use would be associated with reduced MACE risk compared to DPP-4 inhibitors in this high-risk population, consistent with benefits observed in general diabetes populations.

## Materials and Methods

### Study design and data source

This was a retrospective cohort study utilizing the TriNetX US Collaborative Network, a federated health research network providing access to de-identified electronic medical records (EMRs) from large healthcare organizations across the United States [24]. The TriNetX platform aggregates longitudinal patient-level data including demographics, diagnoses (ICD-10-CM codes), procedures (CPT codes), medications (RxNorm, ATC classification), laboratory values, and vital signs. The federated architecture enables researchers to conduct queries across participating institutions while preserving patient privacy through de-identification and aggregate-level data presentation [24].

The analysis was conducted on the US Collaborative Network configuration, which at the time of query execution (March 13, 2026, 00:53:42 UTC) comprised 67 participating healthcare organizations. All 67 healthcare organizations responded to the query, with 51 providers contributing patients to cohort 1 (SGLT2 inhibitor group) and 48 providers contributing to cohort 2 (DPP-4 inhibitor group). This multi-institutional approach enhances generalizability by capturing diverse practice settings, geographic regions, patient populations, and treatment patterns across the United States.

The study protocol adhered to the Strengthening the Reporting of Observational Studies in Epidemiology (STROBE) guidelines for reporting observational cohort studies [25]. As this analysis utilized fully de-identified retrospective data accessed through the TriNetX research platform, institutional review board approval was not required per the Common Rule (45 CFR 46.104(d)(4)), which exempts research involving de-identified data from human subject research oversight. Data use agreements between participating institutions and TriNetX govern appropriate use of network data for research purposes. This study was conducted in compliance with all applicable institutional and national ethical guidelines for research using human subjects’ data and adhered to the principles of the Declaration of Helsinki.

### Study population and cohort definition

#### Inclusion criteria

Cohort 1 (SGLT2 inhibitor group) were adult patients (age ≥ 18 years) meeting all of the following criteria: documented diagnosis of RA using ICD-10-CM codes: M06.9 (RA, unspecified), M06.00 (RA without rheumatoid factor, unspecified site), M06 (other RA), M05 (RA with rheumatoid factor), M06.0 (RA without rheumatoid factor); documented diagnosis of T2DM (ICD-10-CM code E11: T2DM). Prescription of SGLT2 inhibitors (ATC classification code A10BK: SGLT2 inhibitors), including empagliflozin, canagliflozin, dapagliflozin, ertugliflozin, and combination products.

Cohort 2 (DPP-4 inhibitor comparison group) were adult patients meeting the same diagnostic criteria for RA and T2DM, with the following medication criteria: prescription of DPP-4 inhibitors (ATC classification code A10BH: DPP-4 inhibitors), including sitagliptin, saxagliptin, linagliptin, alogliptin, and combination products; MUST NOT have any documented prescription of SGLT2 inhibitors (ATC code A10BK) at any point in their medical record.

#### Exclusion criteria

Patients with index events occurring more than 20 years prior to the analysis date (March 13, 2026); patients lacking sufficient follow-up data after index event; and patients with incomplete demographic or diagnostic information preventing propensity score calculation were excluded.

### Index event and follow-up period definition

The index event was operationally defined as the first documented prescription of the respective medication class (SGLT2 inhibitor for cohort 1, DPP-4 inhibitor for cohort 2) among patients meeting all diagnostic inclusion criteria. The index date represents the calendar date when each patient first received a prescription meeting the index event criteria.

The follow-up period commenced 1 day after the index date to ensure temporal separation between medication initiation and outcome assessment, thereby reducing immortal time bias. Given the real-world nature of this study, no pre-specified end date was imposed; follow-up extended until the earliest of: 1) occurrence of an MACE outcome event, 2) death from any cause, 3) last recorded clinical encounter in the EMR system, or 4) data extraction date (March 13, 2026).

Patients whose index event occurred ≥ 20 years before the analysis date were systematically excluded to focus on contemporary treatment patterns and minimize data completeness issues associated with older records. In the current analysis, zero patients from cohort 1 and zero patients from cohort 2 were excluded based on this temporal criterion.

### Outcome ascertainment

The primary outcome was a composite MACE endpoint, defined as the first occurrence of ANY of the following events during the follow-up period (Table 1).

**Table 1:** MACE Composite Endpoint Components

MACE component	ICD-10-CM code/definition
Acute myocardial infarction	I21 (all subcategories)
Cerebral infarction (ischemic stroke)	I63 (all subcategories)
Heart failure	I50 (all subcategories)
Cardiac arrest	I46 (all subcategories)
Cardiovascular death	Deceased status in demographics

ICD-10-CM: International Classification of Diseases, Tenth Revision, Clinical Modification; MACE: major adverse cardiovascular event.

Outcome events were identified through structured ICD-10-CM diagnostic codes recorded in inpatient, emergency department, or outpatient encounters, as well as mortality records within the EMR. For the deceased component, patients were classified as experiencing cardiovascular death if their record indicated deceased status in the EMR. This definition does not incorporate cause-specific mortality adjudication, as such data are not consistently available across participating institutions, representing a potential source of outcome misclassification.

The composite MACE definition aligns with cardiovascular outcome trial endpoints used in diabetes medication studies, facilitating comparison with published literature [2–4]. Only the first occurrence of any MACE component was counted for the primary time-to-event analysis. Patients with documented MACEs prior to the index date were included in the analysis to reflect real-world treatment patterns, though this approach may introduce prevalent case bias.

### Baseline characteristics and propensity score matching variables

To minimize confounding by indication and baseline risk differences between treatment cohorts, we employed 1:1 propensity score matching using nearest-neighbor matching without replacement [26]. The propensity score represented the predicted probability of receiving SGLT2 inhibitors (versus DPP-4 inhibitors) conditional on measured baseline covariates.

Covariates in the propensity score model encompassed: 1) Demographics including: age at index date (continuous variable, years), sex (male, female), and race/ethnicity (White, Black, or African American, Asian, Hispanic, or Latino, other/unknown); 2) Baseline comorbidities (diagnosed at any time prior to or at index date) including: diseases of the circulatory system (ICD-10-CM I00-I99), hypertensive diseases (ICD-10-CM I10-I15), cerebrovascular diseases (ICD-10-CM I60-I69), pulmonary heart disease and diseases of pulmonary circulation (ICD-10-CM I26-I28), chronic rheumatic heart diseases (ICD-10-CM I05-I09), ischemic heart diseases (ICD-10-CM I20-I25), cardiac arrhythmias (ICD-10-CM I47-I49), diseases of arteries, arterioles, and capillaries (ICD-10-CM I70-I79), chronic kidney disease (ICD-10-CM N18), dyslipidemia (ICD-10-CM E78), and obesity (ICD-10-CM E66); 3) Disease-specific variables including: RA disease duration (calculated from earliest RA diagnosis date to index date), presence of rheumatoid factor (seropositive vs. seronegative RA), diabetes duration (calculated from earliest T2DM diagnosis date to index date), and diabetic complications (neuropathy, nephropathy, retinopathy) coded as E11.x subcategories.

The matching process utilized a caliper width of 0.1 standard deviations of the logit of the propensity score to ensure high-quality matches while maximizing sample size retention [26]. Notably, body mass index, smoking status, specific medication dosages, and RA-specific treatment variables (corticosteroid exposure, DMARD regimens, biologic therapy) were not available as structured, matchable variables in the TriNetX platform and could not be included in the propensity score model. Balance achievement was assessed using standardized mean differences (SMDs), with SMD < 0.10 indicating excellent balance and SMD < 0.20 considered acceptable for most covariates.

### Statistical analysis

#### Descriptive statistics

Baseline characteristics were summarized for both unmatched and matched cohorts. Continuous variables were reported as mean ± standard deviation (SD) and median with interquartile range (IQR). Categorical variables were presented as frequencies and percentages. Between-group differences were assessed using independent *t*-tests for continuous variables and Chi-square tests (or Fisher’s exact test when cell counts < 5) for categorical variables. Statistical significance was set at two-tailed α = 0.05.

SMDs were calculated for all baseline covariates to assess matching effectiveness, with particular attention to variables exhibiting SMD ≥ 0.10 after matching, as these represent potential residual confounding.

#### Primary analysis: risk association

The measure of association analysis quantified the proportion of patients experiencing MACE in each cohort and calculated: 1) Risk = (Patients with outcome)/(Total patients in cohort); 2) Risk difference (RD) = Risk – Risk; 3) Risk ratio (RR) = Risk/Risk; 4) Odds ratio (OR) = (Risk/(1 – Risk))/(Risk/(1 – Risk)).

Ninety-five percent confidence intervals (95% CI) were computed using normal approximation for RD and log transformation methods for RR and OR. Z-statistics and corresponding P-values tested the null hypotheses of no difference (RD = 0, RR = 1, OR = 1).

#### Survival analysis

Kaplan-Meier survival curves were constructed to visualize event-free survival (freedom from first MACE) over time for both cohorts. The survival function S(t) represents the probability of remaining free from MACE at time t following the index date.

Censoring: Patients were censored at the date of their last documented clinical encounter in the EMR if they did not experience an MACE, to account for patients exiting the study cohort due to loss to follow-up, transfer to non-participating healthcare systems, or administrative end of data collection. This censoring approach assumes that censoring is independent of MACE risk conditional on measured covariates.

Median survival time was defined as the time point at which the Kaplan-Meier survival probability dropped below 50%. If the survival curve did not reach 50% during the observation period, median survival was reported as not estimable.

Log-rank test: A non-parametric log-rank test compared the overall survival distributions between cohorts, testing the null hypothesis of no difference in MACE-free survival. The test statistic follows a Chi-square distribution with 1 degree of freedom.

Cox proportional hazards regression: We estimated the HR for MACE comparing SGLT2 inhibitor users to DPP-4 inhibitor users using Cox regression. The HR quantifies the instantaneous risk of MACE in cohort 1 relative to cohort 2 at any given time point, assuming proportional hazards.

The proportional hazards assumption was formally tested using Schoenfeld residuals and a Chi-square goodness-of-fit test. Rejection of the null hypothesis (P < 0.05) indicates violation of the assumption, suggesting that the HR varies over time. In cases of non-proportionality, time-stratified analyses or alternative modeling approaches (e.g., restricted mean survival time) would be considered for more valid effect estimation.

#### Sensitivity and subgroup analyses

Although not presented in detail in the primary results, the following sensitivity analyses were planned: 1) exclusion of patients with MACEs prior to index date (incident MACE analysis); 2) stratification by age groups (< 65 years vs. ≥ 65 years); 3) stratification by sex (male vs. female); 4) stratification by baseline CVD presence; 5) stratification by RA serological status (seropositive vs. seronegative); and 6) time-to-event analysis truncated at minimum follow-up duration to address differential censoring. Safety endpoints (e.g., genital infections, diabetic ketoacidosis, volume depletion, fractures) were not assessed in this analysis and should be evaluated in future studies.

### Software and computational environment

All data extraction, cohort construction, propensity score matching, and statistical analyses were performed using the TriNetX Analytics Platform (TriNetX, LLC, Cambridge, MA), which utilizes R statistical computing environment for backend calculations. Kaplan-Meier curves and Cox regression were executed using survival package algorithms. Two-sided P-values < 0.05 were considered statistically significant throughout.

## Results

### Study population and cohort selection

The initial query identified 823 patients meeting inclusion criteria for cohort 1 (RA + T2DM + SGLT2 inhibitor exposure) and 284 patients for cohort 2 (RA + T2DM + DPP-4 inhibitor exposure without SGLT2 inhibitor use). These patients were distributed across 51 and 48 healthcare provider organizations, respectively, within the TriNetX US Collaborative Network.

After 1:1 propensity score matching, 277 matched pairs (total n = 554) were retained for outcome analysis, representing 33.7% of the SGLT2 inhibitor cohort and 97.5% of the DPP-4 inhibitor cohort being successfully matched. The high matching rate in the DPP-4 cohort reflects the smaller initial sample size and successful identification of comparable SGLT2 inhibitor-treated patients across the propensity score distribution.

### Baseline characteristics before propensity score matching

Table 2 summarizes demographic and clinical characteristics of the unmatched cohorts.

**Table 2:** Baseline Characteristics Before Propensity Score Matching (Unmatched Cohorts)

Characteristic	SGLT2 (n = 823)	DPP-4 (n = 284)	P-value	Std Diff
Demographics				
Age (years), mean ± SD	64.1 ± 11.7	64.5 ± 12.0	0.633	0.033
Age (years), median (IQR)	65	65	-	-
Female sex, n (%)	610 (74.1)	211 (74.3)	0.953	0.004
Male sex, n (%)	213 (25.9)	73 (25.7)	0.953	0.004
White race, n (%)	561 (68.2)	177 (62.3)	0.072	0.123
Baseline comorbidities				
Circulatory system diseases, n (%)	770 (93.6)	238 (83.8)	< 0.001	0.312
Hypertensive diseases, n (%)	736 (89.4)	229 (80.6)	< 0.001	0.248
Cerebrovascular diseases, n (%)	201 (24.4)	50 (17.6)	0.018	0.168
Pulmonary heart disease, n (%)	197 (23.9)	35 (12.3)	< 0.001	0.305
Chronic rheumatic heart disease, n (%)	145 (17.6)	18 (6.3)	< 0.001	0.353

DPP-4: dipeptidyl peptidase-4; IQR: interquartile range; n: number of patients; %: percentage; SD: standard deviation; SGLT2: sodium-glucose co-transporter 2 inhibitors; Std Diff: standardized mean difference.

Pre-matching imbalances: Significant differences were observed in several cardiovascular comorbidities. The SGLT2 inhibitor cohort exhibited higher prevalence of circulatory system diseases (93.6% vs. 83.8%, P < 0.001, SMD = 0.312), hypertensive diseases (89.4% vs. 80.6%, P < 0.001, SMD = 0.248), pulmonary heart disease (23.9% vs. 12.3%, P < 0.001, SMD = 0.305), and chronic rheumatic heart disease (17.6% vs. 6.3%, P < 0.001, SMD = 0.353). Cerebrovascular disease prevalence was also higher in cohort 1 (24.4% vs. 17.6%, P = 0.018, SMD = 0.168).

Notably, age and sex distributions were well-balanced even before matching (P = 0.633 and P = 0.953, respectively), with SMD < 0.05 for both variables. The higher cardiovascular comorbidity burden in the SGLT2 inhibitor group likely reflects preferential prescribing of these agents to patients with established CVD, consistent with guideline-directed therapy recommendations based on cardiovascular outcomes trial evidence [2–4].

### Baseline characteristics after propensity score matching

Table 3 presents characteristics of the propensity-matched cohorts, demonstrating successful balance achievement.

**Table 3:** Baseline Characteristics After Propensity Score Matching (Matched Cohorts, n = 277 per Group)

Characteristic	SGLT2 (n = 277)	DPP-4 (n = 277)	P-value	Std Diff
Demographics				
Age (years), mean ± SD	63.6 ± 11.0	64.4 ± 12.0	0.377	0.075
Age (years), median (IQR)	64	65	-	-
Female sex, n (%)	210 (75.8)	206 (74.4)	0.694	0.033
Male sex, n (%)	67 (24.2)	71 (25.6)	0.694	0.033
White race, n (%)	187 (67.5)	174 (62.8)	0.246	0.099
Baseline comorbidities				
Circulatory system diseases, n (%)	235 (84.8)	238 (85.9)	0.718	0.031
Hypertensive diseases, n (%)	225 (81.2)	229 (82.7)	0.659	0.038
Cerebrovascular diseases, n (%)	34 (12.3)	50 (18.1)	0.058	0.162
Pulmonary heart disease, n (%)	34 (12.3)	35 (12.6)	0.898	0.011
Chronic rheumatic heart disease, n (%)	17 (6.1)	18 (6.5)	0.861	0.015

DPP-4: dipeptidyl peptidase-4; IQR: interquartile range; SD: standard deviation; SGLT2: sodium-glucose co-transporter 2; Std Diff: standardized difference.

Post-matching balance: The propensity score matching successfully eliminated previously observed imbalances. All demographic and comorbidity variables achieved excellent balance with SMD < 0.20 for all characteristics. Notably, circulatory system disease prevalence was now nearly identical (84.8% vs. 85.9%, P = 0.718, SMD = 0.031), as was hypertensive disease (81.2% vs. 82.7%, P = 0.659, SMD = 0.038).

Cerebrovascular disease prevalence showed the largest remaining standardized difference (SMD = 0.162), though this remained within the acceptable threshold (< 0.20) and was no longer statistically significant (P = 0.058). The propensity score density distributions demonstrated substantial overlap after matching, confirming common support and validity of the matching procedure.

### Follow-up duration

Table 4 summarizes follow-up time for both unmatched and matched cohorts.

**Table 4:** Follow-Up Duration Before and After Propensity Score Matching

Cohort	Mean (days)	SD (days)	Median (days)	IQR (days)
Before propensity score matching				
SGLT2 inhibitors (n = 823)	1,275.97	979.45	1,009	1,300
DPP-4 inhibitors (n = 284)	2,361.68	1,430.90	2,325	2,022
After propensity score matching				
SGLT2 inhibitors (n = 277)	1,432.98	1,032.95	1,140	1,489
DPP-4 inhibitors (n = 277)	2,347.93	1,421.38	2,324	2,011

DPP-4: dipeptidyl peptidase-4; IQR: interquartile range; n: number of patients; SD: standard deviation; SGLT2: sodium-glucose co-transporter 2.

Differential follow-up: A notable disparity in follow-up duration persisted even after propensity matching. The DPP-4 inhibitor cohort had substantially longer mean follow-up (2,347.93 days ≈ 6.4 years) compared to the SGLT2 inhibitor cohort (1,432.98 days ≈ 3.9 years), reflecting the more recent market introduction of SGLT2 inhibitors (empagliflozin approved 2014, canagliflozin 2013, dapagliflozin 2014) compared to DPP-4 inhibitors (sitagliptin approved 2006, saxagliptin 2009).

The median follow-up was 1,140 days (approximately 3.1 years) for SGLT2 users versus 2,324 days (approximately 6.4 years) for DPP-4 users. This differential follow-up introduces potential for informative censoring bias and affects interpretation of cumulative risk estimates. The survival analysis with time-to-event methodology partially mitigates this concern by appropriately censoring patients at their last observation, though differential censoring patterns could still influence results through competing risks and time-varying hazards.

### Primary outcome: MACEs

#### Risk analysis

Table 5 presents the primary risk analysis results for the composite MACE endpoint in the propensity-matched cohorts.

**Table 5:** Risk Analysis for Composite MACE Endpoint (Propensity-Matched Cohorts)

Measure	SGLT2 (n = 277)	DPP-4 (n = 277)	Estimate (95% CI)	P-value
Patients with MACE, n	119	135	-	-
Risk (proportion)	0.430 (43.0%)	0.487 (48.7%)	-	-
Risk difference	-	-	-0.058 (−0.141 to 0.025)	0.172
Risk ratio	-	-	0.881 (0.735–1.057)	-
Odds ratio	-	-	0.792 (0.567–1.107)	-

CI: confidence interval; DPP-4: dipeptidyl peptidase-4; MACE: major adverse cardiovascular event; n: number of patients; SGLT2: sodium-glucose co-transporter 2.

Primary findings: Among the 277 propensity-matched pairs, 119 patients (43.0%) in the SGLT2 inhibitor cohort experienced at least one MACE component during follow-up, compared to 135 patients (48.7%) in the DPP-4 inhibitor cohort.

The absolute RD was −5.8 percentage points (95% CI: −14.1% to +2.5%), favoring SGLT2 inhibitors but not reaching statistical significance (z = −1.364, P = 0.172). The RR of 0.881 (95% CI: 0.735–1.057) suggests an approximately 12% relative risk reduction with SGLT2 inhibitors, though the CI crosses the null value of 1.0. Similarly, the OR of 0.792 (95% CI: 0.567–1.107) indicates lower odds of MACE with SGLT2 inhibitor use, again without statistical significance.

Interpretation: While point estimates consistently favor SGLT2 inhibitors, the lack of statistical significance precludes definitive conclusions. The 95% CIs are compatible with risk reductions as large as 14.1 percentage points or risk increases as large as 2.5 percentage points. The study was likely underpowered to detect a clinically meaningful difference, with *post-hoc* power estimates of approximately 40–50% to detect the observed effect size. This substantial risk of type II error, combined with differential follow-up duration affecting cumulative risk estimation, means that the absence of statistical significance should not be interpreted as evidence of no effect. All findings should be considered hypothesis-generating.

#### Kaplan-Meier survival analysis

Table 6 summarizes Kaplan-Meier survival analysis results.

**Table 6:** Kaplan–Meier Survival Analysis for Time to First MACE

Parameter	SGLT2 inhibitors (n = 277)	DPP-4 inhibitors (n = 277)
Patients with MACE, n	119	135
Median survival (days)	2227	2750
Median survival (years)	6.1	7.5
Survival probability at end of follow-up	38.45%	9.50%

DPP-4: dipeptidyl peptidase-4; MACE: major adverse cardiovascular event; n: number of patients; SGLT2: sodium-glucose co-transporter 2.

Median survival time: The median time to first MACE was 2,227 days (approximately 6.1 years) in the SGLT2 inhibitor cohort versus 2,750 days (approximately 7.5 years) in the DPP-4 inhibitor cohort. This seemingly paradoxical finding, shorter median survival in the SGLT2 group despite lower cumulative risk, reflects the differential censoring patterns and distribution of event timing rather than a true adverse effect.

Survival probability at end of observation period: By the end of the observation window, the estimated MACE-free survival probability was 38.45% in the SGLT2 cohort versus 9.50% in the DPP-4 cohort. Importantly, these estimates are not directly comparable due to substantially different follow-up durations and differential censoring patterns between groups. This substantial difference reflects the longer follow-up duration in the DPP-4 group (median 6.4 vs. 3.9 years), allowing more time for events to accumulate. The much lower survival probability in the DPP-4 group (9.50%) indicates that by their median follow-up of 6.4 years, over 90% had experienced MACE, compared to approximately 61% in the SGLT2 group at their median follow-up of 3.9 years.

Log-rank test: The log-rank test comparing overall survival distributions yielded χ^2^ = 3.794 (df = 1, P = 0.051), approaching but not reaching conventional statistical significance (α = 0.05). This borderline P-value should be interpreted with substantial caution; the substantially different follow-up durations (median 3.1 vs. 6.4 years) and differential censoring patterns between groups may independently account for the observed distributional difference in survival curves, without necessarily reflecting a treatment effect. This borderline result should be interpreted cautiously given the methodological limitations, including substantially different follow-up durations between groups and potential for differential censoring bias.

Table 7 presents Cox proportional hazards regression results.

**Table 7:** Cox Proportional Hazards Regression and Proportionality Test

Parameter	Estimate	95% CI	P-value
Hazard ratio (SGLT2 vs. DPP-4)	1.284	0.998–1.652	-
Test for proportionality (χ^2^, df = 1)	4.981	-	0.026

CI: confidence interval; χ^2^: Chi-square statistic; DPP-4: dipeptidyl peptidase-4; df: degrees of freedom; SGLT2: sodium-glucose co-transporter 2.

HR: The Cox regression yielded an HR of 1.284 (95% CI: 0.998–1.652) for SGLT2 inhibitor users compared to DPP-4 inhibitor users. However, this estimate is not validly interpretable because the proportional hazards assumption was violated (see below).

Violation of proportional hazards assumption: The test for proportionality (Schoenfeld residuals test) yielded χ^2^ = 4.981 (df = 1, P = 0.026), indicating significant violation of the proportional hazards assumption. This means the HR is not constant over time; the relative hazard of MACE between groups changes during the follow-up period. This violation invalidates interpretation of the single HR estimate, as it represents an average across time periods where the true HR may vary substantially.

Implications of non-proportionality: This finding suggests that the treatment effect of SGLT2 inhibitors may vary over time. Several scenarios could explain this pattern: Early hazard phase: SGLT2 inhibitors may be associated with slightly higher hazard in the initial period post-initiation (potentially related to volume depletion, acute kidney injury, or metabolic adaptation), followed by protective effects emerging over longer duration as observed in cardiovascular outcome trials [2, 3]. Differential depletion of susceptibles: The DPP-4 cohort’s longer observation time allows depletion of high-risk patients early in follow-up, leaving a healthier survivor cohort, while the SGLT2 cohort includes more patients in their early treatment phase when cardiovascular risk remains elevated. Informative censoring: The substantial difference in censoring patterns between groups (median follow-up 3.1 vs. 6.4 years) violates the Cox model assumption that censoring is non-informative conditional on measured covariates.

Given the proportionality violation, the single HR should be interpreted cautiously and does not represent a valid summary measure of treatment effect. Time-stratified analyses or restricted mean survival time approaches would provide more valid effect estimates but were not available in the primary analysis output.

### Synthesis of findings

Risk analysis (cumulative incidence over entire follow-up): SGLT2 inhibitors associated with 5.8 percentage point lower absolute risk of MACE (43.0% vs. 48.7%), though not statistically significant (P = 0.172). Kaplan-Meier analysis (time-to-event, accounting for censoring): Log-rank test P = 0.051 did not reach statistical significance and is substantially confounded by differential follow-up duration between groups; it does not independently support a treatment effect. Cox HR: HR = 1.284 (95% CI: 0.998–1.652) is non-interpretable due to significant violation of the proportional hazards assumption (Schoenfeld residual test P = 0.026) and substantial differential follow-up between groups.

The most interpretable finding is the risk analysis showing numerically lower cumulative MACE incidence with SGLT2 inhibitors, which does not achieve statistical significance but shows a consistent directional effect across RD, RR, and OR point estimates. The Cox model results are compromised by assumption violations and differential follow-up, limiting their reliability as a summary measure of treatment effect.

## Discussion

### Principal findings

This real-world evidence study examining cardiovascular outcomes in patients with concurrent RA and T2DM revealed a non-significant trend toward lower MACE risk among SGLT2 inhibitor users compared to DPP-4 inhibitor users. After propensity score matching for baseline demographics and comorbidities, the SGLT2 inhibitor cohort experienced an absolute risk reduction of 5.8 percentage points (43.0% vs. 48.7% cumulative MACE incidence), corresponding to an OR of 0.792 (95% CI: 0.567–1.107, P = 0.172).

While this difference did not reach statistical significance, the consistent directional effect across multiple risk metrics (RD, RR, OR) and the consistent direction of point estimates across risk metrics represents a hypothesis-generating signal given the established cardioprotective effects of SGLT2 inhibitors in general T2DM populations [2–4, 18]. The borderline log-rank result (P = 0.051) does not reach statistical significance and is substantially confounded by the differential follow-up duration; it should not be interpreted as an independent signal of treatment benefit. These findings are hypothesis-generating and require confirmation in adequately powered prospective studies.

### Comparison with existing literature

The magnitude of cardiovascular risk reduction observed in this study (approximately 12% relative risk reduction based on RR = 0.881) is numerically smaller than reported in landmark cardiovascular outcomes trials of SGLT2 inhibitors in general T2DM populations. The EMPA-REG OUTCOME trial demonstrated a 14% reduction in three-point MACE (HR 0.86, 95% CI 0.74–0.99) and a 38% reduction in cardiovascular death (HR 0.62, 95% CI 0.49–0.77) with empagliflozin versus placebo [2, 14]. Similarly, the CANVAS program showed a 14% reduction in MACE (HR 0.86, 95% CI 0.75–0.97) with canagliflozin [3, 15].

However, direct comparisons are challenging due to fundamental differences in study design, population characteristics, and comparator groups. The present study employed an active comparator (DPP-4 inhibitors) rather than placebo, which would be expected to attenuate observed effect sizes since DPP-4 inhibitors themselves demonstrate cardiovascular safety and potential modest benefits in some populations [22, 23]. Furthermore, the unique inflammatory milieu and immunosuppressive medication exposures in RA patients may modulate SGLT2 inhibitor efficacy differently than in general T2DM populations through effects on inflammatory pathways, endothelial function, and cardiovascular remodeling [7, 8].

Recent meta-analyses confirm that SGLT2 inhibitors significantly reduce all-cause mortality (OR 0.82, 95% CI 0.75–0.90), cardiovascular mortality, myocardial infarction, and hospitalization for heart failure across diverse patient populations [18, 19]. These consistent findings across multiple trials, populations, and settings support the biological plausibility of SGLT2 inhibitor cardiovascular effects as a drug class; however, the present study did not demonstrate statistically significant cardiovascular benefit in the RA-T2DM population and should not be interpreted as confirmatory evidence of this drug class’s effects in this specific population.

Limited prior data exist specifically examining SGLT2 inhibitor outcomes in RA patients with diabetes. The intersection of chronic inflammatory disease and metabolic dysregulation represents a critical evidence gap, as RA populations have been systematically excluded or underrepresented in landmark cardiovascular outcome trials [2–4]. Observational studies have demonstrated that RA patients with concurrent diabetes or insulin resistance experience the highest cardiovascular risk, with HRs exceeding 2.5 compared to the general population [6], highlighting the urgent need for evidence-based glucose-lowering strategies in this vulnerable population.

### Mechanistic considerations in RA populations

If SGLT2 inhibitors do confer cardiovascular benefit in RA populations, several pathophysiological mechanisms beyond glucose lowering could be involved.

First, SGLT2 inhibitors demonstrate anti-inflammatory effects, including reductions in pro-inflammatory cytokines, oxidative stress markers, and inflammatory cell activation [18, 20]. These effects may be particularly relevant in RA, where chronic systemic inflammation drives accelerated atherosclerosis and cardiovascular risk independent of traditional risk factors [7–9].

Second, SGLT2 inhibitors improve hemodynamic parameters through natriuresis, blood pressure reduction, and preload/afterload reduction [18, 20]. These effects may benefit RA patients, who frequently have subclinical cardiac dysfunction, diastolic abnormalities, and heart failure with preserved ejection fraction related to chronic inflammation and fibrosis.

Third, SGLT2 inhibitors provide renal protective effects, slowing progression of chronic kidney disease and reducing albuminuria [4, 18]. These renal benefits may be particularly valuable in RA patients, who experience increased rates of chronic kidney disease related to disease activity, medication nephrotoxicity, and comorbid hypertension.

Fourth, SGLT2 inhibitors alter myocardial substrate utilization, increasing ketone body production and improving myocardial energetics [20]. This metabolic shift may benefit the chronically inflamed and metabolically stressed myocardium in RA patients.

Finally, SGLT2 inhibitors reduce body weight and visceral adiposity, potentially decreasing adipokine dysregulation and insulin resistance that contribute to cardiovascular risk in RA populations [13, 18].

The interplay between SGLT2 inhibitor mechanisms and RA-specific pathophysiology warrants dedicated mechanistic studies examining inflammatory biomarkers, endothelial function, cardiac imaging parameters, and disease activity measures in RA patients treated with SGLT2 inhibitors.

### Strengths and limitations

#### Strengths

This study possesses several methodological strengths that enhance validity and generalizability.

First, the large federated health research network encompassing 67 healthcare organizations provides generalizable real-world evidence across diverse practice settings, geographic regions, patient populations, and treatment patterns [24].

Second, rigorous propensity score matching incorporating comprehensive baseline comorbidities and demographics successfully balanced measured confounders, as evidenced by SMDs < 0.20 for all covariates post-matching [26].

Third, by restricting to patients with dual diagnoses of RA and T2DM, the study addresses a clinically important high-risk population with limited evidence to guide glucose-lowering agent selection.

Fourth, the active comparator design using DPP-4 inhibitors reflects real-world clinical decision-making and minimizes confounding by indication related to diabetes severity, as both classes represent second- or third-line therapy options with established cardiovascular safety profiles [21, 23].

Fifth, the composite MACE endpoint aligned with established cardiovascular trial endpoints enhances comparability with published literature [2–4].

Sixth, the study utilized contemporary data extending through 2026, capturing recent treatment patterns and outcomes.

Finally, the analysis employed multiple complementary statistical approaches (risk analysis, Kaplan-Meier survival analysis, Cox regression) to comprehensively characterize cardiovascular outcomes.

#### Limitations

Several important limitations warrant consideration in interpreting these findings.

First, the observational design precludes definitive causal inference despite propensity matching. Unmeasured confounders including RA disease activity indices (DAS28, CDAI), inflammatory markers (erythrocyte sedimentation rate (ESR), C-reactive protein (CRP)), specific DMARD regimens, corticosteroid dosing patterns, socioeconomic factors, lifestyle variables, medication adherence, and treatment switching patterns may bias results [7, 8].

Second, differential follow-up duration represents a critical limitation, with median follow-up of 3.1 years for SGLT2 users versus 6.4 years for DPP-4 users. This disparity reflects SGLT2 inhibitors’ more recent market availability and introduces potential for differential censoring, varying opportunity for event accrual, and time-varying hazards. The violation of the proportional hazards assumption (P = 0.026) is likely associated with this differential follow-up pattern and further limits interpretation of survival analysis results.

Third, limited sample size following propensity matching (277 pairs) likely resulted in insufficient statistical power to detect modest-to-moderate effect sizes with statistical significance, as evidenced by wide confidence intervals crossing the null. *Post-hoc* power calculations suggest approximately 40–50% power to detect the observed effect size, indicating substantial risk of type II error.

Fourth, the heterogeneous MACE composite endpoint includes cardiovascular death ascertained through deceased status without specific cause-of-death validation, potentially introducing misclassification. Heart failure events, myocardial infarction, stroke, and cardiovascular death carry different biological mechanisms and may respond differentially to SGLT2 inhibitor therapy, as demonstrated in cardiovascular outcome trials showing particularly robust effects on heart failure and cardiovascular death [2, 3, 4].

Fifth, violation of proportional hazards in Cox regression (P = 0.026) indicates time-varying treatment effects not adequately captured by a single HR, limiting interpretability of survival analysis results. Time-stratified analyses or restricted mean survival time approaches would provide more valid effect estimates but were not available in the primary analysis.

Sixth, medication adherence and switching could not be rigorously assessed using administrative data. Real-world medication adherence, discontinuation, and switching between drug classes may dilute true per-protocol effects toward the null, underestimating treatment efficacy.

Seventh, unmeasured RA-specific variables represent a major source of residual confounding. Disease activity scores (DAS28, CDAI), inflammatory biomarkers (CRP, ESR), specific biologic and targeted synthetic DMARD use (TNF inhibitors, IL-6 inhibitors, JAK inhibitors), corticosteroid exposure and dosing patterns, and extra-articular manifestations were not captured in the propensity score model. These variables are strongly associated with both cardiovascular risk and treatment selection, and their absence substantially limits causal interpretation of the observed associations [7, 8].

Eighth, coding accuracy represents an inherent limitation of observational studies using administrative data. Reliance on ICD-10-CM diagnostic codes introduces potential for misclassification, though validation studies of administrative diabetes and RA diagnoses generally show high positive predictive value exceeding 85–90%. Finally, prevalent case inclusion (patients with MACEs prior to the index date were not excluded) may introduce prevalent-user bias and complicate interpretation of time-to-event outcomes. A sensitivity analysis excluding patients with prior MACEs would strengthen causal interpretation but was not conducted in this analysis due to platform limitations, and should be prioritized in future studies.

### Clinical and research implications

The findings of this study, while not definitive due to limitations discussed above, provide hypothesis-generating data suggesting a possible directional association between SGLT2 inhibitor use and lower MACE incidence compared to DPP-4 inhibitors in patients with concurrent RA and T2DM. Given the established cardiovascular benefits of SGLT2 inhibitors in general diabetes populations [2, 3, 4, 18] and the high cardiovascular risk inherent to RA [5, 6, 9], these findings do not yet support preferential use of SGLT2 inhibitors over DPP-4 inhibitors solely based on cardiovascular outcomes in this population. However, given the established cardiovascular benefits of SGLT2 inhibitors in general T2DM populations, clinicians may consider SGLT2 inhibitors in appropriate candidates with concurrent RA and T2DM, particularly those with: established CVD or multiple cardiovascular risk factors, heart failure with reduced or preserved ejection fraction, chronic kidney disease with or without albuminuria, high RA disease activity with systemic inflammation, need for additional blood pressure, weight, or glycemic control.

These recommendations align with current diabetes management guidelines from the American Diabetes Association, which recommend SGLT2 inhibitors (or GLP-1 receptor agonists) as preferred agents in patients with T2DM and established atherosclerotic CVD, heart failure, or chronic kidney disease, independent of baseline HbA1c or individualized HbA1c target [27].

However, clinicians must also consider potential safety concerns in RA populations, including increased risk of genital mycotic infections (which may be enhanced by immunosuppressive therapies), diabetic ketoacidosis (particularly with corticosteroid use or acute illness), volume depletion and hypotension (especially with diuretics or during acute inflammation), and potential drug interactions with immunosuppressive agents. Shared decision-making incorporating patient preferences, comorbidities, and RA-specific factors should guide therapeutic choices.

### Future research priorities

This study highlights several critical research priorities to optimize cardiovascular outcomes in RA patients with T2DM.

Prospective randomized controlled trial (RCT): A dedicated RCT comparing SGLT2 inhibitors to active control (DPP-4 inhibitor or sulfonylurea) in RA patients with T2DM, adequately powered for MACE outcomes with standardized follow-up duration of 3–5 years. Stratification by RA disease activity, DMARD regimen, and baseline CVD would enable subgroup analyses.

Mechanistic studies: Investigation of SGLT2 inhibitor effects on inflammatory biomarkers (CRP, IL-6, TNF-α), endothelial function, arterial stiffness, and cardiac imaging parameters (echocardiography, cardiac MRI) in RA populations. Assessment of potential effects on RA disease activity scores and synovial inflammation would clarify immunomodulatory properties beyond glucose lowering.

Long-term safety surveillance: Comprehensive assessment of SGLT2 inhibitor-associated risks including genital mycotic infections, diabetic ketoacidosis, volume depletion, fractures, and amputations specifically in RA populations receiving concomitant immunosuppressive therapies. Registry studies with extended follow-up would enable rare adverse event detection.

Heart failure-focused studies: Given robust heart failure benefits in cardiovascular outcome trials [17], focused analysis of heart failure hospitalization, progression, and phenotype (preserved vs. reduced ejection fraction) in RA patients warrants dedicated investigation. RA patients experience increased heart failure risk independent of ischemic heart disease, suggesting distinct pathophysiology that may respond to SGLT2 inhibitor mechanisms.

Kidney outcome studies: Evaluation of renal protective effects in RA-associated kidney disease and diabetic nephropathy overlap syndromes. Assessment of albuminuria, eGFR slope, and progression to end-stage renal disease would clarify renal benefits in this population.

Real-world effectiveness studies: Pragmatic effectiveness studies using electronic health records, claims databases, and registries to assess cardiovascular outcomes, medication adherence, treatment persistence, and safety in diverse RA populations receiving SGLT2 inhibitors. Comparative effectiveness research against GLP-1 receptor agonists, another class with established cardiovascular benefits, would inform treatment sequencing.

Health economics analysis: Cost-effectiveness modeling of SGLT2 inhibitor use in RA-diabetes populations considering cardiovascular event reduction, quality-adjusted life years, medication costs, and healthcare utilization. Budget impact analyses would inform health system decision-making.

Biomarker studies: Identification of biomarkers predicting SGLT2 inhibitor response in RA populations, including baseline inflammatory markers, genetic variants, metabolomic profiles, and cardiac biomarkers. Precision medicine approaches may enable targeted therapy to maximize benefit-risk ratio.

### Conclusion

In this real-world cohort of patients with RA and T2DM, SGLT2 inhibitor use was associated with a numerically lower but statistically non-significant risk of MACEs compared to DPP-4 inhibitors (43.0% vs. 48.7%; OR 0.792, 95% CI 0.567–1.107, P = 0.172). The Cox proportional hazards model was non-interpretable due to violation of the proportional hazards assumption (P = 0.026) and substantial differential follow-up (median 3.1 vs. 6.4 years). These results are hypothesis-generating and do not constitute evidence of cardiovascular benefit. While these findings do not establish cardiovascular superiority of SGLT2 inhibitors in RA populations, they are consistent with the known cardioprotective profile of this drug class in general T2DM populations. Prospective, adequately powered trials with comprehensive adjustment for RA-specific confounders (disease activity, DMARD use, corticosteroid exposure) and standardized follow-up are warranted to determine whether SGLT2 inhibitors provide cardiovascular benefit in patients with concurrent RA and diabetes.

## Data Availability

The authors declare that data supporting the findings of this study are available within the article. The data that support the findings of this study are derived from the TriNetX US Collaborative Network and are available in aggregated and de-identified form within the TriNetX platform. Restrictions apply to the availability of these data, which were used under license for this study and are not publicly available; however, access may be obtained upon reasonable request to TriNetX and with permission of the participating healthcare organizations.

## Abbreviations

ATC: Anatomical Therapeutic Chemical
CDAI: Clinical Disease Activity Index
CI: confidence interval
CKD: chronic kidney disease
CRP: C-reactive protein
CPT: Current Procedural Terminology
CVD: cardiovascular disease
DAS28: Disease Activity Score in 28 joints
DMARD: disease-modifying antirheumatic drug
DPP-4: dipeptidyl peptidase-4
EMR: electronic medical record
ESR: erythrocyte sedimentation rate
GLP-1: glucagon-like peptide-1
GIP: glucose-dependent insulinotropic polypeptide
HbA1c: hemoglobin A1c
HR: hazard ratio
ICD-10-CM: International Classification of Diseases, Tenth Revision, Clinical Modification
IL-1β: interleukin-1 beta
IL-6: interleukin-6
IQR: interquartile range
IRB: Institutional Review Board
ITOM: Information Technology and Operations Management
JAK: Janus kinase
JBI: Joanna Briggs Institute
MACEs: major adverse cardiovascular events
MI: myocardial infarction
NLRP3: NOD-, LRR- and pyrin domain-containing protein 3
OR: odds ratio
RA: rheumatoid arthritis
RCT: randomized controlled trial
RR: risk ratio
SD: standard deviation
SGLT2: sodium-glucose co-transporter 2
SMD: standardized mean difference
STROBE: Strengthening the Reporting of Observational Studies in Epidemiology
T2DM: type 2 diabetes mellitus
TNF-α: tumor necrosis factor-alpha
US: United States
